# Medulloblastoma in a 13-Year-Old Female: A Comprehensive Case Report

**DOI:** 10.7759/cureus.66359

**Published:** 2024-08-07

**Authors:** Khizer Ansari, Shilpa A Gaidhane, Pratapsingh Parihar, Iram Saifi, Azeem I Saifi

**Affiliations:** 1 Medicine and Surgery, Jawaharlal Nehru Medical College, Datta Meghe Institute of Higher Education and Research, Wardha, IND; 2 Epidemiology and Public Health, Jawaharlal Nehru Medical College, Datta Meghe Institute of Higher Education and Research, Wardha, IND; 3 Radiodiagnosis, Jawaharlal Nehru Medical College, Datta Meghe Institute of Higher Education and Research, Wardha, IND; 4 Radiology, Jawaharlal Nehru Medical College, Datta Meghe Institute of Higher Education and Research, Wardha, IND

**Keywords:** posterior fossa tumours, brainstem glioma, medulloblastoma, neurology, mr spectroscopy

## Abstract

The majority of children's brain cancers are posterior fossa tumours, which include brainstem gliomas, medulloblastomas (MBs), juvenile pilocytic astrocytomas, and ependymomas. This report details a 13-year-old female presenting with headache, nausea, and ataxia. With typical magnetic resonance imaging (MRI) and magnetic resonance spectroscopy (MRS) results, the MRI indicated a solid lesion in the fourth ventricle, producing obstructive hydrocephalus. Pilocytic astrocytoma, ependymoma, MB, and other conditions are examples of differential diagnoses. In addition to underscoring the need for early intervention to enhance prognosis and outcomes for paediatric patients with posterior fossa tumours, the case highlights the vital role that sophisticated imaging plays in early detection and therapy.

## Introduction

Medulloblastoma (MB) is a highly aggressive tumour originating from embryonic cells. It usually affects youngsters and is located in the vermis area of the cerebellum [1]. With an incidence rate of 0.05 per 100,000 per year, this tumour is uncommon in adults, yet it makes up 30% of all paediatric central nervous system (CNS) neoplasms. Children under 10 are diagnosed with MB in around 75% of instances [2]. Posterior fossa tumours in children are infratentorial, comprising 45-60% of all brain tumours in the paediatric age group. They include MB, juvenile pilocytic astrocytoma, ependymoma, and brainstem glioma. Atypical teratoid rhabdoid tumours and hemangioblastoma also appear in the differential diagnosis [3].

On magnetic resonance imaging (MRI), the presence of solid and cystic components, mural nodules, enhancement characteristics, calcifications, origin, and plasticity of the tumour help to make the diagnosis. With the help of magnetic resonance spectroscopy (MRS), MR perfusion imaging also assists in confirming the diagnosis. Various clinical features, especially headaches in children and cerebellar signs and symptoms, highlight the need for diagnosis using advanced diagnostic techniques like MRI, particularly in children.

## Case presentation

A 13-year-old female was brought to the emergency room by her relatives with chief complaints of headache for two weeks, which increased on straining and was associated with nausea and vomiting. The patient also complained of dizziness, vertigo, and ataxia. On examination, the pulse was 68 bpm, and the blood pressure was 140/90 mmHg. There was no icterus, pallor, or lymphadenopathy. Examination of the cardiovascular, respiratory, musculoskeletal, and gastrointestinal systems was normal. The patient had no history of any comorbidities and was not on any medications. The patient was advised to undergo an MRI of the brain with contrast to rule out the cause of the headache.

**Figure 1:**
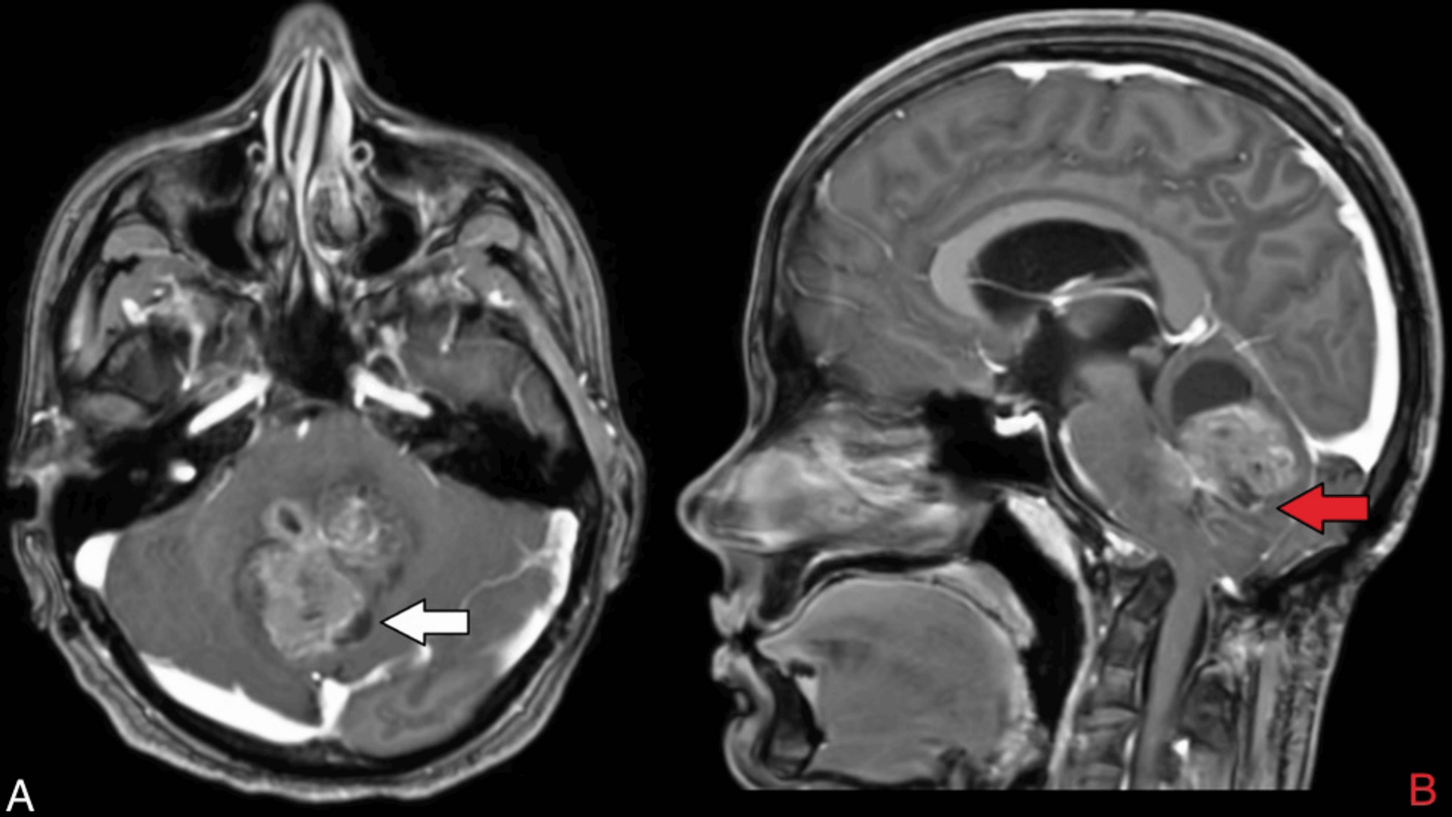
MRI brain T1 contrast axial and sagittal images A) The white arrow shows a heterogeneous signal intensity solid lesion with few pseudo-cystic areas in the posterior fossa seen involving the vermis and roof of the fourth ventricle, bilateral cerebellar peduncles and causing a mass effect in the form of perilesional oedema and anterior displacement of the brain stem. B) The red arrow shows compressing the vermis, pons, and medulla, leading to upstream dilatation of the third and bilateral lateral ventricles. MRI: Magnetic resonance imaging

**Figure 2:**
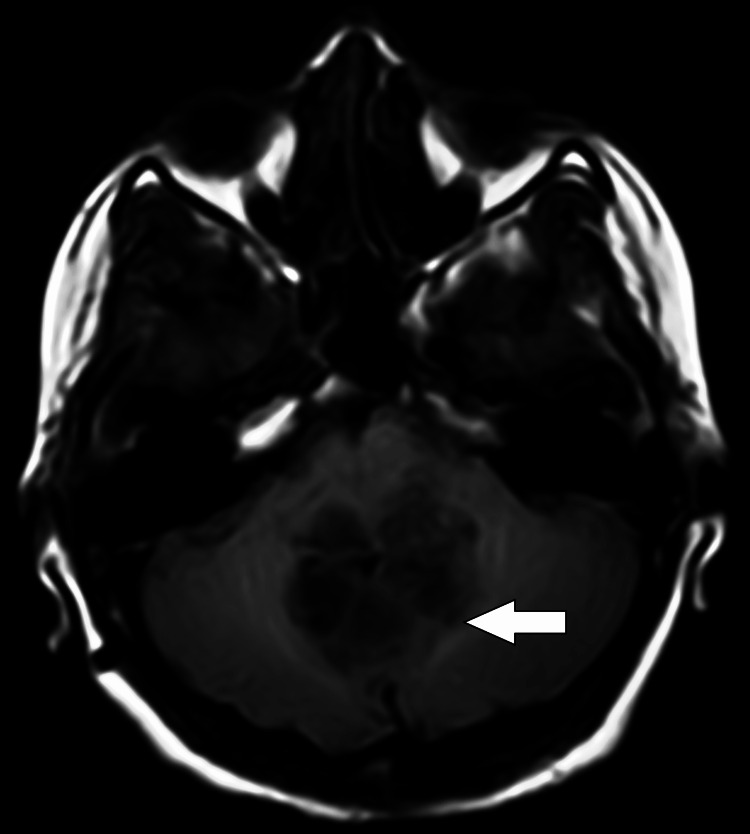
Axial T1 weighted brain MRI The white arrow shows the lesion appears predominantly hypo-intense. MRI: Magnetic resonance imaging

**Figure 3:**
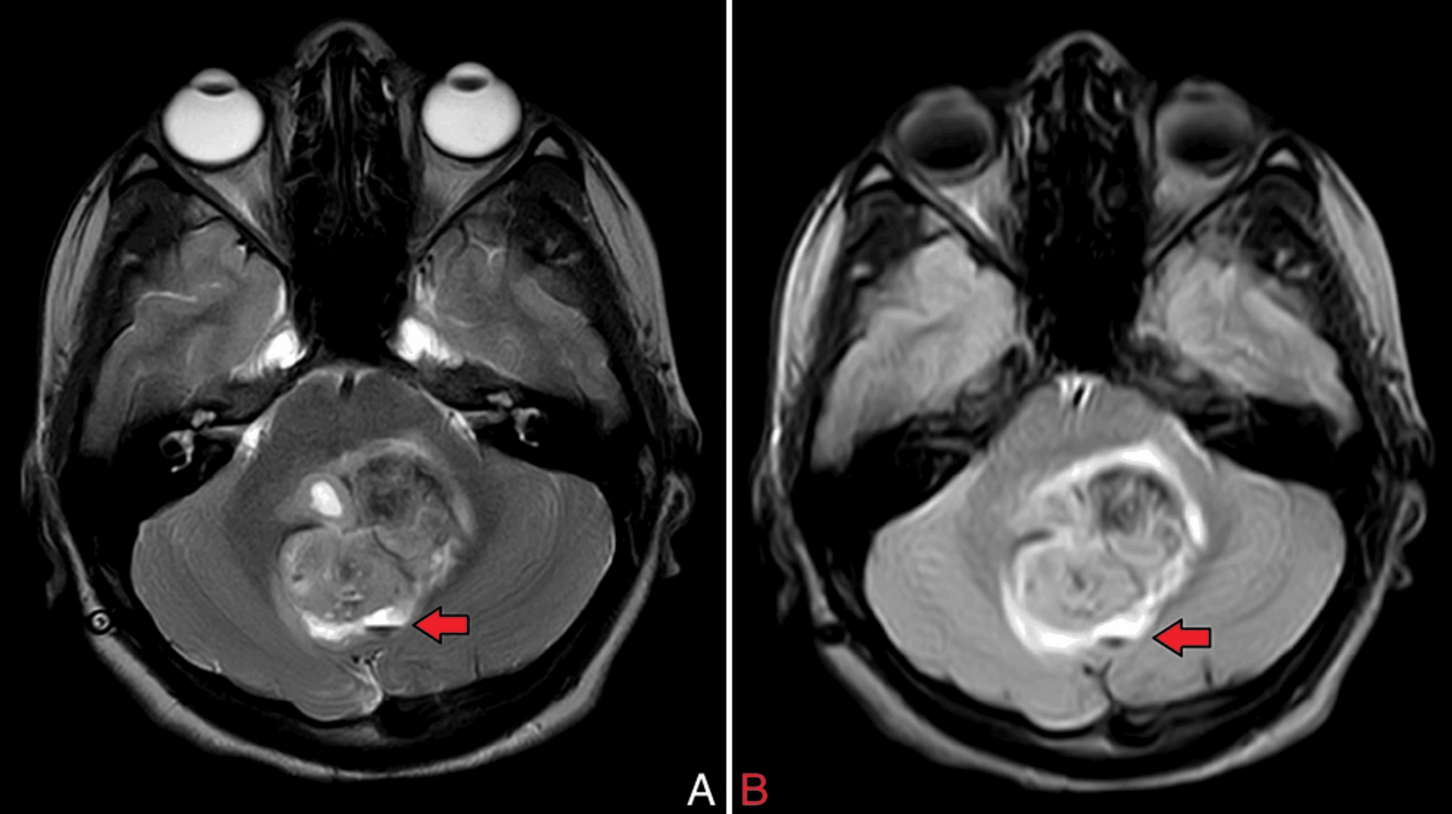
A) Axial T2 and B) FLAIR MRI brain images The red arrow shows that the lesion appears hyperintense with perilesional oedema. FLAIR: Fluid-attenuated inversion recovery; MRI: Magnetic resonance imaging

**Figure 4:**
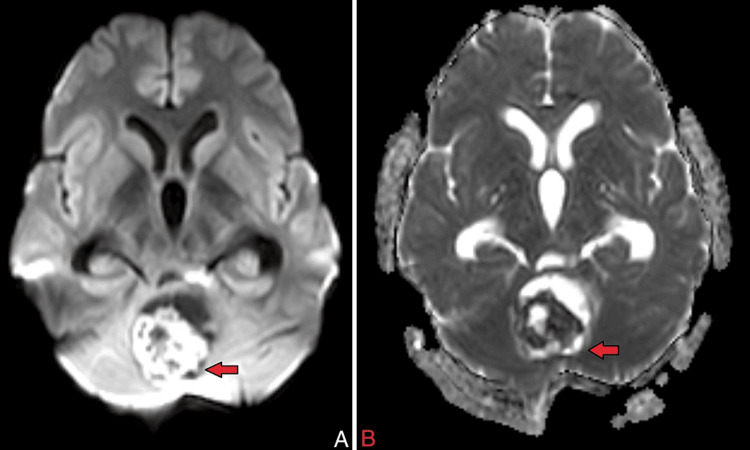
A) Axial DWI and B) ADC MRI brain images The red arrow shows that the lesion restricts DWI and corresponds to a low signal on ADC. DWI: Diffusion-weighted imaging; ADC: Apparent diffusion coefficient; MRI: Magnetic resonance imaging

**Figure 5:**
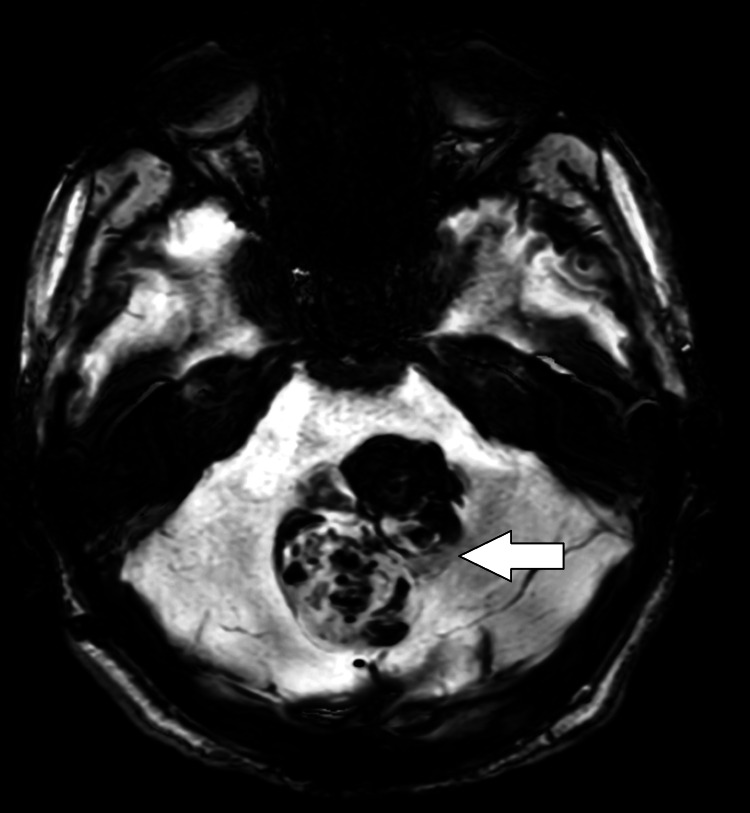
Axial SWI image showing the presence of blooming in the lesion (white arrow). SWI: Susceptibility-weighted imaging

**Figure 6:**
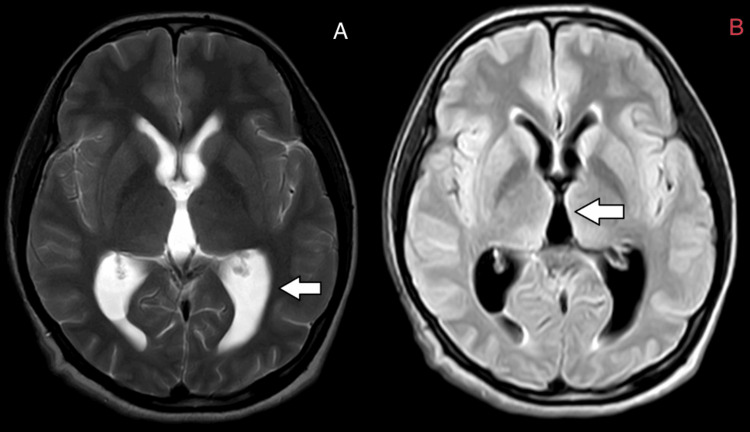
Axial T2 and FLAIR MRI brain images A) Arrow showing dilatation of the lateral ventricle; B) Arrow showing dilatation of the third ventricle. FLAIR: Fluid-attenuated inversion recovery; MRI: Magnetic resonance imaging

**Figure 7:**
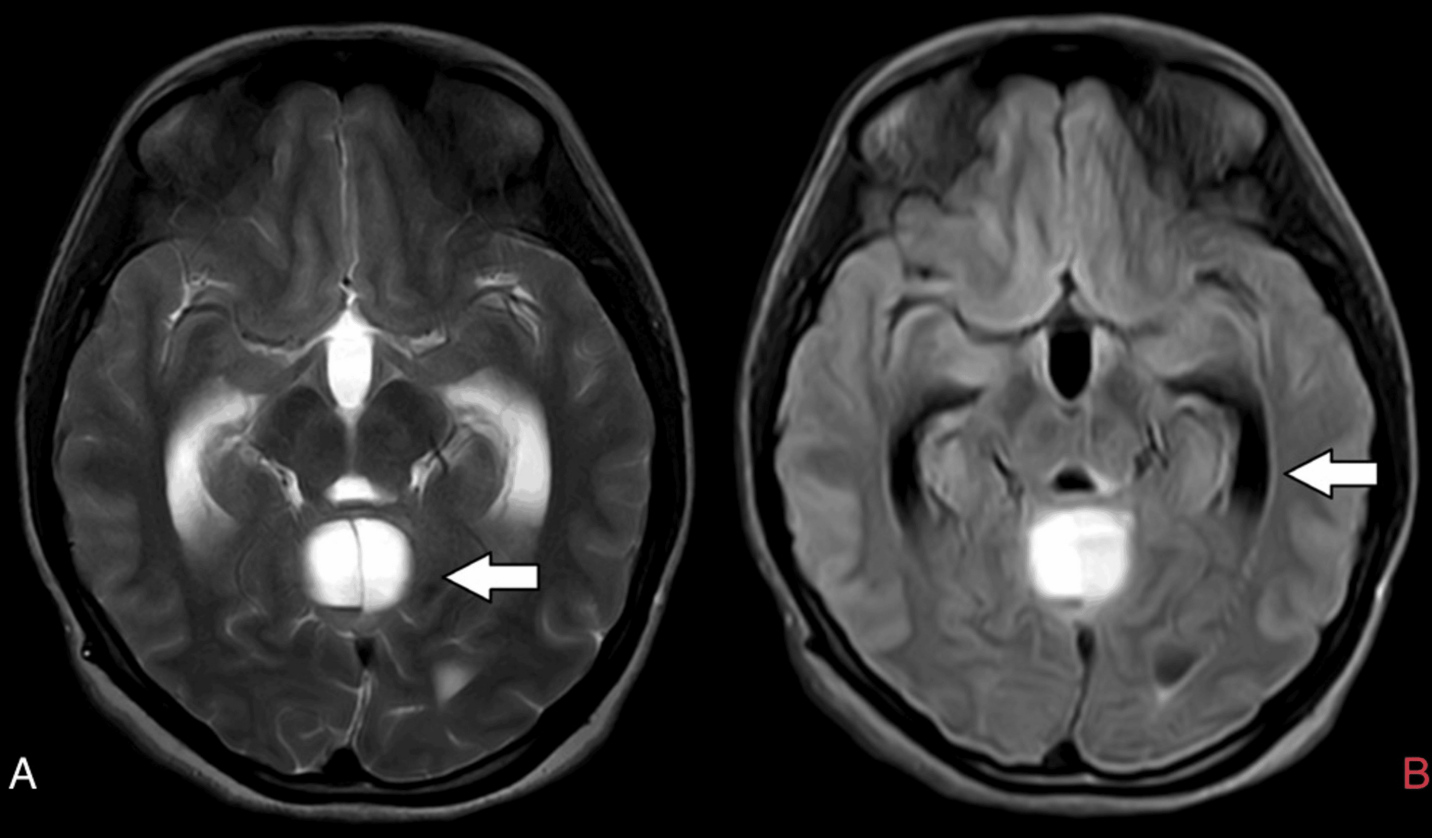
Axial T2 and FLAIR MRI brain images A) Arrow shows dilatation of the fourth ventricle; B) Arrow shows dilatation of the temporal horn of the lateral ventricle. FLAIR: Fluid-attenuated inversion recovery; MRI: Magnetic resonance imaging

**Figure 8:**
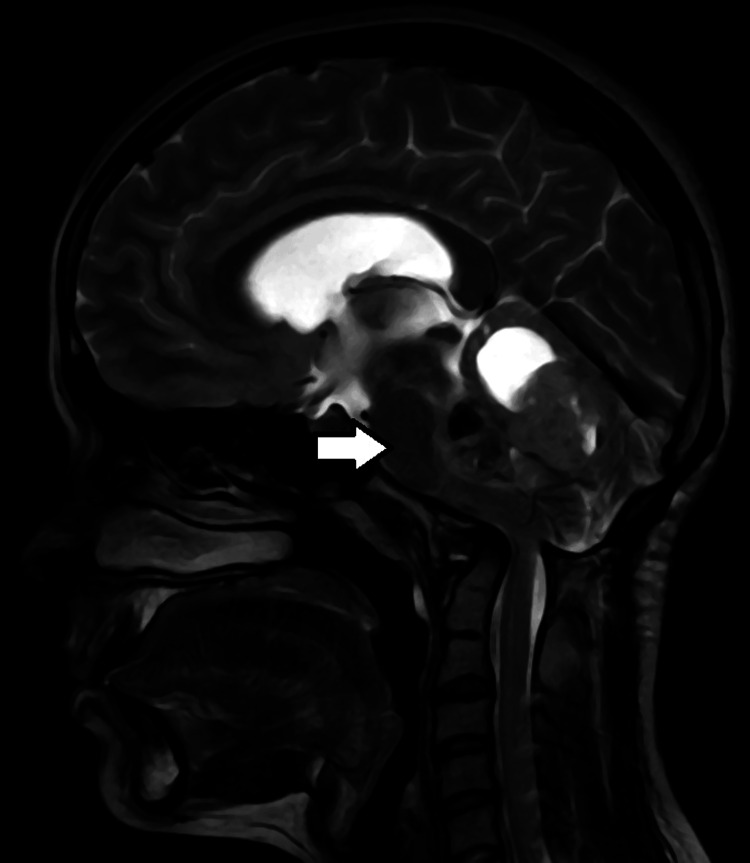
Sagittal T2 MRI brain image The white arrow shows the mass effect of the lesion in the form of anterior displacement of the brain stem. MRI: Magnetic resonance imaging

**Figure 9:**
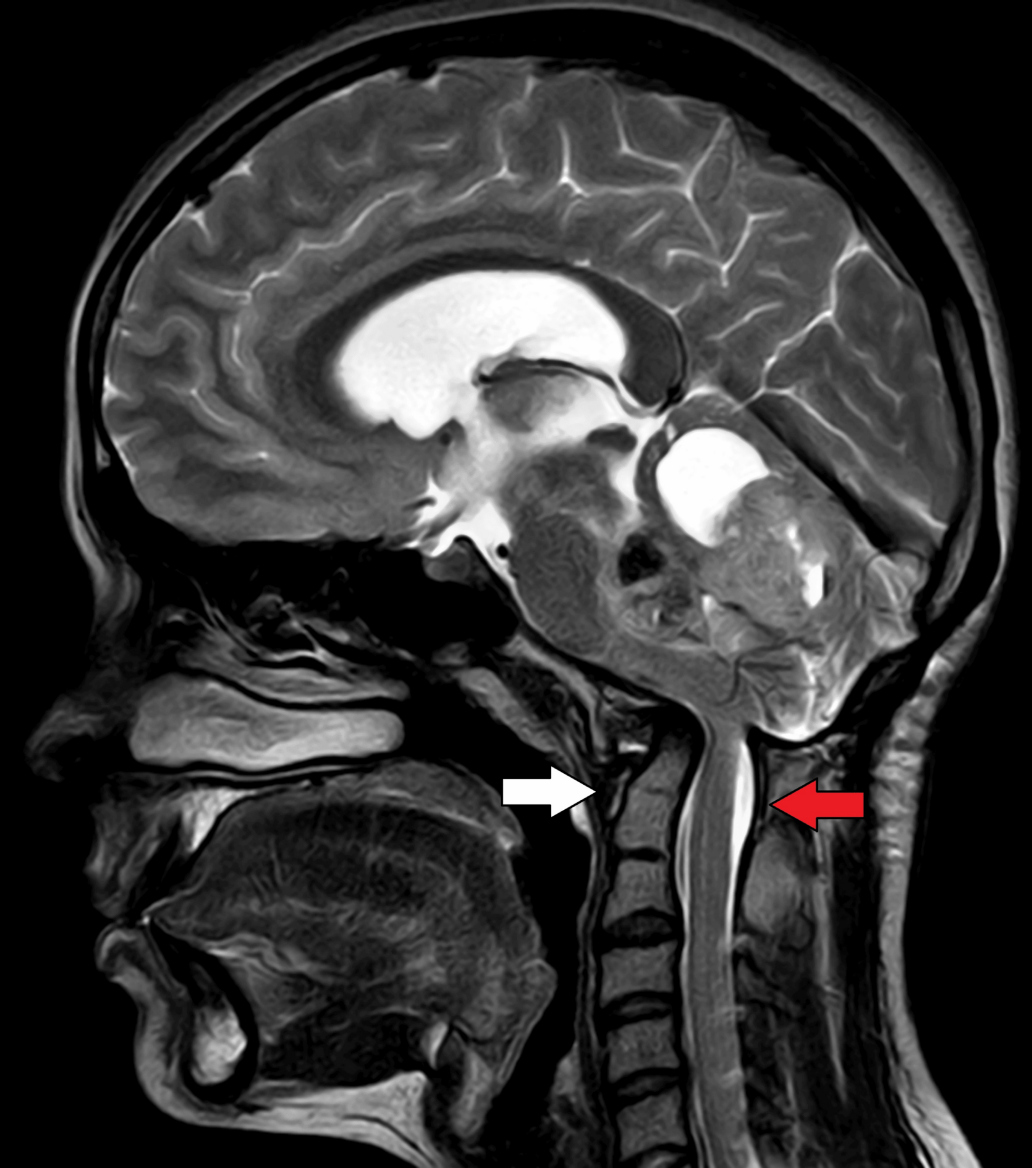
Sagittal MRI brain image The red arrow shows partial assimilation of the occipital bone with C1 vertebra, cervical-medullary kinking with no evidence of oedema. The white arrow shows narrowing is noted at the level of the foramen of magnum with crowding within due to basilar invagination. MRI: Magnetic resonance imaging

On MRI of the brain with contrast, there was evidence of a heterogeneously enhancing, altered signal intensity solid lesion with a few pseudo-cystic areas, predominantly in the region of the vermis and roof of the fourth ventricle. This lesion involved the bilateral middle cerebellar peduncles (left > right), the dorsolateral midbrain on the left side, and caused a mass effect in the form of perilesional oedema, anterior displacement of the brain stem, and compression of the vermis, pons, and medulla (Figures 1, 8). The lesion appeared hypo-intense on T1 (Figure 2), iso to hyperintense on T2/fluid-attenuated inversion recovery (FLAIR) (Figure 3), showed restriction on diffusion-weighted imaging (DWI) and corresponding low signal on apparent diffusion coefficient (ADC) (Figure 4), and showed blooming on susceptibility-weighted imaging (SWI) (Figure 5). The lesion was also causing a mass effect on the fourth ventricle, leading to upstream dilatation of the third and bilateral lateral ventricles, suggestive of obstructive hydrocephalus with periventricular ooze (Figures 6, 7). The lesion measured approximately 4.3 x 4.1 x 3.9 cm. There was evidence of partial assimilation of the occipital bone with the C1 vertebra. Cervico-medullary kinking was noted. There was no evidence of oedema in the present scan. The narrowing was noted at the level of the foramen magnum with crowding due to basilar invagination. There was an increased atlantoaxial interval (Figure 9).

Management includes surgery, chemotherapy, and radiotherapy. The surgical approach for this case involves a midline suboccipital craniotomy and decompression of the lesion. This is followed by radiotherapy, which uses high-energy rays to target and kill any remaining cancer cells that cannot be surgically removed. Radiotherapy helps eliminate microscopic cancer cells that might still be present in the brain or spinal cord, reducing the risk of the tumour recurring. Subsequently, chemotherapy is administered, using drugs that circulate throughout the body to kill cancer cells, which is crucial for targeting cells that have spread beyond the primary tumour site. By utilizing this three-part approach - surgery, radiation, and chemotherapy - we can improve outcomes and increase survival rates.

## Discussion

There are various posterior cranial fossa tumours, which include MB, ependymoma, astrocytoma, hemangioblastoma, and cerebellar metastasis. MBs are common posterior cranial fossa tumours. They can cause mass effects on the fourth ventricle and create obstructive hydrocephalus. They are more common in males. On histology, they are small, round, and blue cell tumours.

On MRI, they are commonly seen to arise from the vermis and extend from the fourth ventricle through its roof to the brain stem [4]. They appear heterogeneously hyperintense and can show cysts and necrotic areas; sometimes, calcifications and perilesional oedema are also found [5,6]. The lesion can show restricted diffusion because of high cellular content and low ADC values [7]. They show elevated choline and decreased N-acetylaspartate (NAA) peaks on MRS.

On computed tomography (CT), they can appear as a heterogeneously hyperdense midline mass in the posterior fossa, most likely arising from the vermis or roof of the fourth ventricle, which is formed by the superior medullary velum, and extending into the fourth ventricle and brainstem, creating a mass effect in the form of obstructive hydrocephalus. Treatment of this tumour includes surgery, radiotherapy, and chemotherapy, and to decrease hydrocephalus, ventriculoperitoneal (VP) shunt placement is needed. The prognosis of the tumour depends on the molecular subtype; however, if the diagnosis is at an early age of fewer than three years, if cerebrospinal fluid (CSF) metastases are present, and when resection of the tumour is not complete, these factors can show poor prognosis [8].

The differential for posterior fossa tumours includes ependymoma, which commonly originates from the fourth ventricle floor and can extend into the foramen of Luschka. Calcification is more common in ependymoma. They show plasticity in nature, which means they can extend wherever they find space to grow [9].

Other differentials include pilocytic astrocytoma, which contains mural nodules showing intense enhancement and cystic areas [10]. Another differential includes atypical teratoid rhabdoid tumours, which are more common in infants and younger children. They mimic MBs and can only be distinguished with the help of immunohistochemical markers [11]. Another differential can be brainstem gliomas, but most of them do not show intense enhancement. Gliomas, which are diffuse in nature, also do not have restricted diffusion. Comparatively, they have higher ADC values than MBs. Brainstem gliomas are usually low-grade [12].

Another differential includes hemangioblastoma, which is found chiefly in middle-aged adults [13]. It can be associated with von Hippel-Lindau syndrome. On MRI, it can appear as a solid tumour or as a large cystic tumour with a mural nodule. Hemangioblastomas can have high vascularity and a high relative cerebral blood volume (rCBV) value on perfusion MRI [14]. Cerebellar metastasis is more common in elderly individuals, and a primary tumour is needed before it can be confirmed. However, single posterior fossa metastasis is rare in children.

## Conclusions

This case report highlights the importance of posterior fossa tumours in children, which includes a multilevel diagnostic approach with the help of advanced technologies like CT, MRI with contrast, MRS, MR perfusion imaging, and histopathological diagnosis. These methods not only aid in diagnosis but also assist in management and provide clues about the tumour's prognosis. Early detection of headache complaints in children using non-ionizing modalities, such as MRI, facilitates early diagnosis and treatment. This case report also provides an approach to distinguish different posterior fossa tumours in children. Early diagnosis using MRI helps in treatment and allows society to make informed and practical decisions for the health of young children and their future prospects.
